# Supplementary material for: Are carbon emissions trading and green financial instruments synergistic? -Comprehensive quantitative research based on content analysis
Source: PLoS One. 2024 Mar 7;19(3):e0298601. doi: 10.1371/journal.pone.0298601 (PMC10919601; doi:10.1371/journal.pone.0298601)
Supplement: S1 Data — (ZIP) [file pone.0298601.s001.zip › Data/地级市Z_F工作报告.zip!/║■▒▒/╛ú╓▌╩╨/└┤╫╘╣1⁄2╓┌║┼í╛┬φ┐╦╩2╛▌═°í┐_2016.docx]

各位代表：

现在，我代表市人民政府向大会报告工作，请予审议，并请各位政协委员和其他列席人员提出意见。

一、“十二五”时期经济社会发展回顾

“十二五”时期，是荆州发展史上极不平凡的时期。五年来，面对复杂多变的外部环境和宏观经济持续下行的严峻形势，在省委、省政府和市委的坚强领导下，在市人大、市政协的监督支持下，我们紧紧依靠全市人民，推进“五路壮腰”，奋力赶本赶超，主要经济指标在全省的位次、份额全面提升，增幅跻身并保持第一方阵，实现了“十二五”圆满收官。

--这五年，是经济实力明显提升的五年。

工业经济增量提质。规模以上工业企业由649家增加到1141家、净增492家；规模以上工业增加值年均增长14.7%。主营业务收入10亿元以上的工业企业达到27家、净增15家，福娃集团、中兴能源产值突破百亿元；税收过千万的企业达到112家、净增73家。农产品加工、装备制造、医药化工产值分别达到1500亿元、490亿元和270亿元，11个产业纳入省重点扶持成长型产业集群。

农业基础进一步巩固。粮食产量“十二连增”，我市荣获“全国粮食生产先进市”称号，监利县、洪湖市、公安县荣获“全国粮食生产先进县”称号，石首市被授予“全国蔬菜标准化建设示范县”，松滋市获批国家级农产品质量安全创建试点县市。淡水产品、油菜籽产量保持全国市州第一，洪湖市、公安县成为全国首批渔业健康养殖示范县。华中农高区累计投入64.2亿元，获批国家农业科技园、国家农业科技创新与集成示范基地、国家现代农业科技示范区。新型农业经营主体由2.2万个增加到4.1万个，数量居全省市州之首。土地整理和农业综合开发完成投资51.3亿元，为建国以来投入最多的五年。

现代服务业增长较快。万达、绿地、卓尔、世纪金源等一批知名城市综合体进驻荆州，限上商贸企业由387家增加到917家。电子商务高速增长，石首市、监利县分获全国、全省电子商务进农村示范县市。古城修复与保护世行贷款项目获得批准，纪南生态文化旅游区纳入省级战略。投资60亿元的华强文化项目落户荆州，九老仙都、周老嘴革命旧址群等景区建成开放，投资15亿元的关公义园即将开园。旅游总收入由52.3亿元增加到166亿元，年均增长26%。浦发、平安、汉口、交通、中信、招商等股份制银行到荆州布点，菲利华在深圳创业板IPO首发，能特科技重组上市，索瑞电器等5家企业参与上市企业并购重组，金科环保等4家企业在“新三板”挂牌，“四板”挂牌托管登记企业达到36家。金融机构贷款余额突破千亿元，连续8年被评为省“A级信用市州”。

--这五年，是发展后劲明显增强的五年。

基础设施建设加快。沪蓉高铁通车，彻底改变了中心城区没有客运铁路的历史。江南高速通车，潜石高速荆州段、武汉城市圈环线荆州段、东卷高速建成，江北高速、沙公高速加快建设。蒙华铁路全线开工，荆州长江公铁大桥合龙，石首长江大桥开工。荆松一级公路即将通车，荆当旅游公路、318国道城区段改线工程开工。全市96%以上的乡镇通达二级以上公路。荆江航道整治一期工程完工，引江济汉工程建成通航，荆州组合港项目建设加快。荆南四河堤防加固、荆江大堤综合整治等工程顺利推进。城乡电力改造、通信网络设施建设力度加大。“十二五”时期，累计完成交通投资493.5亿元、水利投资103亿元，分别为“十一五”时期的3.5倍和2倍。

开放开发取得新进步。纳入国家老工业基地调整改造规划试点城市，成功获批国家级承接产业转移示范区，荆州开发区晋升为国家级经济技术开发区，洪湖市与武汉经济技术开发区合作共建新滩工业园，全市开发区实际开发面积达到251.9平方公里，是“十一五”时期的1.7倍。累计招商引资实际到位资金3930亿元，利用外资5.3亿美元，外贸出口51.5亿美元。引进亿元以上工业项目833个，华电江陵电厂、荣成再生科技、香港家居产业园等116个10亿元以上项目落户荆州，华讯方舟、五方光电、高田汽配、恒隆转向器、凯乐光纤、长利玻璃、宜化肥业等项目建成投产，成为新的经济增长点。

科技创新力度加大。新增校企共建研发中心9家、省级工程技术研究中心11家、院士专家工作站34家。申请国家专利6900项，登记省级科技成果453项，获省级以上科技奖励114项。高新技术企业由47家增加到102家，预计产值由63.5亿元增加到132亿元。恒隆集团、四机厂分别获得长江质量奖、长江质量奖提名奖。新增中国驰名商标15件、国家地理标志证明商标24件，新增注册商标8804件。我市被授予“全国科技进步考核先进市”称号，荆州区、松滋市、公安县获评“全国科技进步考核先进县”。

--这五年，是城乡面貌明显变化的五年。

中心城区形象改观。成功获批国家园林城市、国家智慧城市试点城市、省级卫生城市。累计投入城建资金73.6亿元，新建、改建和拓宽城市主次干道60条、124.7公里，深圳大道、上海大道、太湖大道、农高大道、天谷大道、庄王大道建成通车。沙北、荆北、商务片区建设加快推进，新图书馆、公共检验检测平台、荆州中学和沙市中学新校区、荆州市中心医院迁建工程开工建设，荆州火车站、市体育中心成为城市新地标。新建明月、临江仙、玉桥、范家渊和荆襄河湿地“五大公园”，升级中山、文湖等5个公园和20个街头游园，建成荆沙河、西干渠两个带状公园，新增绿地面积314公顷。资源集成、重心下沉的大城管格局基本形成，城市管理水平逐步提升。

城乡统筹步伐加快。松滋市、公安县通过国家园林城市评审，松滋市、石首市、监利县创成省级文明城市，监利县、洪湖市被评为省级卫生城市。建制镇总规和村庄规划基本实现全覆盖，松滋市洈水镇等乡镇被纳入全国改革重点镇，荆州区弥市镇等13个乡镇成功创建国家重点镇，公安县斗湖堤镇等5个乡镇入选“全省百强乡镇”。沙市区岑河镇、石首市新厂镇、江陵县三湖农场等11个“四化同步”发展试点镇建设加快，“福娃模式”成为全省城乡一体化发展的典型。新建通村公路5145公里，农村饮水安全工程新增受益人口210万人。连续开展五轮“三万”活动，农村基础条件得到较大改善。

生态建设扎实推进。全面开展“绿满荆州”行动，市域森林覆盖率达到20%，松滋市、公安县、石首市被评为全国绿化模范县市，洪湖湿地晋升为国家级自然保护区，环荆州古城、洈水、崇湖、三菱湖、菱角湖成功申报国家湿地公园。节能减排阶段性目标任务完成。饮用水源地专项整治、中心城区内河补水、农村环境连片整治等工程有效实施，村级垃圾清运体系实现全覆盖。“三禁两治”工作持续深化，空气质量得到改善。成功获批国家生态文明先行示范区。

--这五年，是民生福祉明显增进的五年。

社会保障全面加强。累计民生支出1037亿元，占一般公共财政支出的79.6%。荣获“全国创业先进城市”称号，累计新增城镇就业41.6万人，农村劳动力转移就业27.8万人。社会保险参保人数达到384.6万人，城乡居民社会养老保险实现全覆盖，连续11次提高企业退休人员养老金。城市低保标准由每月300元逐步提高到480元，农村低保标准由每年1440元提高到3000元。累计完成保障性安居工程13.7万套，改造农村危旧房4.1万户。连续五年，政府承诺为民办理的实事圆满完成。

社会事业协调发展。“两基”教育通过国家评估验收，新、改、扩建学校508所，组建荆州职业教育集团，荆州区、松滋市、公安县、洪湖市通过县域义务教育均衡发展国家督导认定。成功承办湖北省第十四届运动会，建成国家级综合性高水平体育后备人才基地。县级公立医院综合改革稳步推进。以村为单位人畜血吸虫感染率控制在1%以下。基层公共文化服务设施建设全覆盖，在全省率先建成农村智能广播网，文化广电、新闻出版事业进一步发展。石首“跳三鼓”纳入国家级非遗代表性项目名录，荆州博物馆获评首批国家一级博物馆，熊家冢被授予全省唯一国家考古遗址公园。大力培育和践行社会主义核心价值观，加强精神文明建设，荆州电视台、市工商局、市气象局等单位被评为全国文明单位，传导正能量的《好人开讲》节目引起社会共鸣。积极参与“东方之星”号游轮翻沉事件救援及善后处置工作，荆州广大干部群众身上体现的“大义大爱”精神，受到党中央国务院、省委省政府的高度肯定和社会各界的一致好评。国防动员、人民防空、民兵预备役工作得到加强，荣获全国“双拥”模范城市称号，以荆州命名的军舰入列东海舰队。计划生育、扶贫开发、对口支援、外事侨务、台湾事务、民族宗教、防震减灾、残疾人事业、机关事务管理等工作取得新进步，统计、物价、供销、烟草、盐务、农垦、移民、社科、史志、档案、保密和无线电等工作取得新成绩。

社会治理能力提升。公共服务向基层延伸，城乡网格化管理基本实现全覆盖。平安荆州、法治荆州建设强力推进，立体化治安防控体系基本建立，治安案件万人发案率保持全省最低，公众安全感、治安满意度和执法满意度大幅提升。信访积案化解成效显著，信访秩序依法规范，信访总量逐年下降。公共安全体系不断完善，食品药品、道路交通、安全生产专项整治力度加大，应急管理工作全面加强，安全生产形势持续稳定。

--这五年，是政府自身建设明显加强的五年。

政务效能逐步提高。行政审批制度改革不断深化，“一表通、一站式、一条龙”服务模式全面铺开，企业注册登记“三证合一”在全省率先实施，行政审批事项调整为291项。文物旅游、卫生计生、文化广电、教育体育等“大部制”改革顺利完成。市环科所、市房产估价所等一批事业单位转企改制。完成不动产登记职责整合，全省第一本不动产权证书在我市颁发。

依法行政能力提升。认真执行市人大及其常委会决议决定并向其报告重大工作，主动与市政协协商讨论重大事项，自觉接受法律监督、民主监督和社会监督，五年共办理人大代表建议、批评和意见452件，政协建议案、提案1531件。密切联系工会、共青团、妇联等群众团体。“六五”普法全面完成，我市被赋予地方政府规章制定权、人大立法权。

政风行风得到改善。扎实推进“党的群众路线教育实践活动”和“三严三实”专题教育，认真贯彻落实中央八项规定精神、省委六条意见和市委七条意见，深入开展“治庸问责”“转作风、抓落实、晒实绩”等活动。强化效能监察和审计监督，着力整治违规多占住房、超标办公用房、违反财经纪律等“四风”突出问题，清理“吃空饷”439人，腾退超标办公用房1.5万平方米，“三公”支出大幅下降。

各位代表！刚刚过去的2015年，我们认真落实党中央、国务院和省委、省政府稳增长各项政策措施，经济社会发展呈现“稳中有进、进中趋优”的良好态势。预计实现地区生产总值1590.5亿元、增长8.5%。完成固定资产投资1950.5亿元、增长18.1%；社会消费品零售总额946.1亿元、增长12.8%；地方公共财政预算收入103.9亿元、增长10.8%；城乡居民人均可支配收入达到25382元、13728元，分别增长9.8%、8.7%。

各位代表！我市“十二五”发展成绩来之不易，这是省委、省政府和市委正确领导的结果，是全市上下群策群力、艰苦奋斗的结果。在此，我代表市人民政府，向全市广大干部群众，向人大代表、政协委员、各民主党派、工商联、无党派人士和人民团体，向驻荆部队、武警官兵、政法干警和部省垂管单位，向所有关心支持荆州建设与发展的海内外朋友，表示衷心的感谢并致以崇高的敬意！

在总结成绩的同时，也要清醒地认识到我市经济社会发展中还存在一些困难和问题。主要表现在：受宏观经济持续下行影响，经济发展增速放缓，地区生产总值、社会消费品零售总额、外贸出口等指标未达预期目标；经济总量还不大，产业集聚度还不高，县域经济还不强；生产要素约束日趋加剧，结构性矛盾比较突出，转型发展面临压力较大；地方财力仍较薄弱，基本公共服务供给不足，城乡极少数群众生活依然贫困；行政审批等改革进展不快，制约市场主体活力发挥的制度成本仍然较高；面对新常态，少数公务人员不愿作为、不敢作为、不会作为的问题不同程度存在。对此，我们一定要更加重视，认真加以解决。

二、“十三五”时期经济社会发展主要目标和任务

“十三五”时期，是我市全面建成小康社会的决胜期，也是实施壮腰工程“十年大振兴”的攻坚期。综合分析经济形势，我们仍然处在大有可为、大有作为的发展阶段。

一方面，有利发展的大好机遇正在汇聚。为适应经济新常态，国家优化空间发展布局，加快推进供给侧结构性改革和新型城镇化；省委、省政府高度重视荆州壮腰工程。荆州作为全省唯一同时跻身长江经济带、长江中游城市群、洞庭湖生态经济区和国家级承接产业转移示范区四大国家战略的市州，上级的支持力、外界的注意力和改革释放的新红利，正在叠加转化为加快发展的新动力。我们务必牢牢抓住机遇、精准对接机遇、用足用好机遇，把当前的战略机遇期变成后发赶超的黄金发展期。

另一方面，有利发展的基础优势更加彰显。实施壮腰工程以来，固定资产投资、规模以上工业企业销售收入、地方公共财政预算收入实现“三年翻番”；地区生产总值、固定资产投资、农产品加工业产值、招商引资实际到位资金、金融机构贷款余额实现“五个过千亿”，这些都为“十年大振兴”奠定了坚实基础。更为可贵的是，壮腰工程战略汇聚了荆江两岸的民心士气，激发了广大干部的激情斗志，全市上下已经形成争先创优的发展氛围。只要我们保持战略定力，努力进位赶超，就一定能够迎来“十三五”时期的快发展、大发展、优发展。

根据《中共荆州市委关于制定荆州市国民经济和社会发展第十三个五年规划的建议》，市政府制定了《荆州市国民经济和社会发展第十三个五年规划纲要（草案）》，提请大会审议。

今后五年政府工作的指导思想是：高举中国特色社会主义伟大旗帜，全面贯彻党的十八大、十八届三中、四中、五中全会和省委十届七次全会精神，以马克思列宁主义、毛泽东思想、邓小平理论、“三个代表”重要思想、科学发展观为指导，深入贯彻习近平总书记系列重要讲话精神，围绕“四个全面”战略布局荆州实施，积极落实创新、协调、绿色、开放、共享发展理念，坚持人民主体地位，坚持依法治市，坚持扩大开放，坚持“竞进提质、升级增效、以质为帅、量质兼取”，把握“三维纲要”，推进“四化同步”，深化“五路壮腰”，加快推进壮腰工程“十年大振兴”，与全省同步在中部地区率先全面建成小康社会，打造湖北经济增长“第四极”，努力将荆州建设成为江汉平原现代化中心城市和长江中游重要的中心城市。

今后五年的主要发展目标是：全市生产总值年均增长9%以上，规模以上工业增加值年均增长12%以上，全社会固定资产投资年均增长16%以上，社会消费品零售总额年均增长13%以上，外贸出口年均增长12%以上，地方公共财政预算收入年均增长10%以上，城乡居民人均可支配收入分别年均增长9.5%、10%以上。力争2020年地区生产总值跃上3000亿元台阶，主要指标平衡协调，总量比2010年增加1.5倍以上，增幅保持全省第一梯队，份额占比继续提高。

今后五年的主要工作思路是：

（一）打造更有支撑力的大产业。用好国家级承接产业转移示范区金字招牌，加快培育食品加工、装备制造、医药化工三个千亿产业和轻工建材、纺织服装、电子信息三个五百亿产业，推动工业增量提质。积极引进和培育高新技术产业和战略性新兴产业，努力形成新的经济增长极。加快转变农业发展方式，大力发展现代农业，推动粮经饲统筹、农林牧渔结合、种养加一体、一二三产业融合发展。以华中农高区为龙头，依托省级农产品加工园和省级农业产业化示范园，大力发展农产品加工业，力争农产品加工产值与农业总产值之比达到3:1。推进质量强市和品牌强市建设，鼓励企业争创精品名牌，全面提升“荆州制造”的市场竞争力。以文化旅游为突破口，大力发展商贸物流、现代金融、信息服务等，推动服务业向个性化和高品质转变。实施“互联网+”行动计划，创建电子商务示范城市。

（二）打造更有竞争力的大城市。积极融入“一带一路”、长江中游城市群和洞庭湖生态经济区战略，加强与“两圈两带”和周边城市的联动合作，推进优势互补、区域协同、融合互通。坚持以规划为龙头，注重向建设和管理两端发力，不断完善城市功能，提升城市品位。按照“一核五极”区域发展模式，统筹谋划“一江两岸”开放开发，致力打造区域性中心城市。建设城市快速通道，推进荆州开发区与江陵滨江新区、沙市经济开发区相向融合发展，将华中农高区核心区纳入中心城区统筹发展，打造新兴产业集聚区、农业高新区和新型煤电煤化基地。实施网络强市战略，加快信息基础设施更新换代，实现公共场所免费WiFi全覆盖；推进数据资源开放共享，落实网络提速降费，打造江汉平原数据中心和后台服务基地，加快建设智慧荆州。深入开展精神文明创建活动，基本建成公共文化服务体系，着力提升城市文化软实力。发展壮大县域经济，支持松滋、公安、石首、监利、洪湖加快建设中等城市。坚持以产业化带动城镇化，构筑生产空间集约高效、生活空间宜居适度、生态空间山清水秀的新型城镇体系，支持有条件的重点镇建设县域副中心，力争全市常住人口城镇化率达到61%。

（三）打造更有吸引力的大环境。加快形成“双十字”铁路网络、“三横七纵一环九桥”高速通道、“一港十区”水运网络，建成荆州民用机场和通用航空机场，打造长江中游区域性综合立体交通枢纽。坚持把绿色发展的责任扛在肩上，统筹推进“蓝天、碧水、净地”工程，综合治理大气污染、水污染和农村面源污染，强化节能减排，发展绿色、循环、低碳经济，加快创建生态市。重点推进荆江大堤、荆南四河、洪湖分蓄洪区东分块、四湖流域防洪排涝灌溉工程建设和洪湖湿地生态功能恢复，统筹实施中心城区、县市城区防洪排涝工程建设和环境保护专项行动，全面提升城乡水利、生态安全保障水平。进一步深化各项改革，加强法治政府建设，加快落实“权力清单”“责任清单”等制度，为更多市场主体蓬勃发展创造条件。完善普惠性政策扶持体系，建立适应创新链需求的科技、金融、人才等各类要素服务体系和产业孵化中心，推动大众创业、万众创新。

（四）打造更有凝聚力的大民生。坚持以人民为中心，把实现好、维护好、发展好广大人民的根本利益作为政府工作的出发点和落脚点，不断增强人民群众的获得感。建成覆盖城乡的公共就业服务体系，保障充分就业、稳定就业。实施居民收入倍增计划，促进城乡居民收入增长高于经济增长。健全覆盖城乡居民的社会保障体系，实现应保尽保。健全住房保障供应体系，全面放开养老服务市场，逐步实现“住有所居、老有所养”。完善城乡社会救助体系，发展社会福利和慈善事业。推进惠及全民的公平教育，建立完善公共体育服务体系，加快建设教育强市、体育强市。深化医药卫生体制改革，促进优质医疗资源下沉。加大血防综合治理力度，实现血吸虫传播阻断目标。实施“三年扶贫攻坚行动”，推进精准扶贫、精准脱贫，确保省定贫困乡镇、贫困村和贫困户与全市人民同步迈入全面小康社会。推进城市基础设施和公共服务向农村延伸，改善农村人居环境，建设美丽乡村。实施食品安全战略，严格安全生产监管，进一步加强和创新社会治理，维护社会和谐稳定。

三、努力实现“十三五”良好开局

2016年，是“十三五”的开局之年，也是壮腰“五年大跨越”的决胜之年。今年经济社会发展的预期目标是：地区生产总值增长9%以上，规模以上工业增加值增长9%以上，固定资产投资增长17%以上，社会消费品零售总额增长13%，外贸出口增长12%以上，地方公共财政预算收入增长11%，城乡居民人均可支配收入分别增长9%、9.5%以上，城镇登记失业率控制在4.5%以内，完成省下达的节能减排任务。

今年要以五大发展理念为引领，以供给侧结构性改革为重点，突出抓好以下工作：

（一）加大产业培育力度。

筑牢工业底盘。统筹安排重大产业发展基金、生产调度资金等政府性资金，设立长江经济带荆州壮腰工程产业基金，引导农产品加工、石油机械、汽车零部件、医药化工、纺织服装等传统产业技改扩能，支持新能源汽车、海洋工程装备、智能制造、生物医药、新材料、电子信息、节能环保等战略性新兴产业发展壮大，推进困难企业兼并重组、资产重组、产业重组。认真落实中央系列减费、降税、松绑“组合拳”政策和促进工业经济稳增长的有关措施，鼓励福娃、中兴能源、白云边向国内行业龙头迈进，支持楚源、沙隆达、凯乐、美的、四机、恒隆、拍马、钢管厂等企业冲刺百亿元产值目标。充分发挥市县两级担保平台、“政银保”、“助保贷”、续贷周转金、小额贷款保证保险等融资杠杆作用，重点支持成长型、科技型中小微企业“小进规”，力争新增规模以上企业150家。探索建立“1+X”大开发区管理模式，实现市县园区共管、政策共享、税收分成，推进产业集聚发展、错位发展。

增强农业优势。以市场农业为导向，加快农业结构调整，提升农业规模化、特色化、品牌化水平，继续创建“百亿斤粮食大市”，打造“荆州味道”精品农产品公共品牌。探索建立农业担保基金，继续抓好农产品加工“四个一批”工程，支持荆州区、松滋市争创省级农产品加工园区，力争产值过百亿的园区达到5个以上，力争全市农产品加工业产值新增200亿元。加快华中农高区产业项目布局，积极创建“中国绿谷”。支持监利县建设国家现代农业示范区。全面落实中央强农惠农富农政策。完成农村土地经营权确权登记颁证，推进农村土地规范有序流转，引导大中专毕业生、返乡人员等领办合作社和家庭农场，支持农业产业化龙头企业通过直接投资、参股经营、签订长期合同等方式，带动农民发展多种形式的适度规模经营。

提升现代服务业。推动服务业发展提速、比重提升、结构优化，确保今年社会消费品零售总额突破千亿元。坚持把文化旅游业作为战略性支柱产业来培育，突出抓好荆州古城景区、楚王车马阵景区、华强文化项目和洪湖、洈水、天鹅洲、荆江南北闸等文化旅游景区创建，整合旅游资源，打造旅游品牌，力争把荆州建设成为有吸引力的旅游目的地，确保旅游总收入增长20%以上。继续实施服务业“五个一百”工程，支持沙市区省级服务业试点示范区建设；加强城市核心商圈建设，推动一批商贸业、服务业主体“个转企”“小进限”；健全和完善农村市场体系。启动县域金融工程和市域金融中心建设，打造集融资担保、融资租赁、产业（基金）投资、股权融资、债权融资等多种融资模式于一体的综合性金融服务平台，壮大支柱产业，服务中小企业，积极引导企业上市、挂牌。支持电商加快发展，重点抓好“淘宝·特色中国·荆州馆”“中国绿谷网”“供销e家”“裕农网”等电商平台建设和服务，力争电商服务村级全覆盖。积极发展现代物流、研发设计、文化创意、人力资源、咨询评估、健康养老和体育休闲等服务业。

（二）千方百计扩大有效投资。

注重招商实效。强化“专业机构、专业队伍、专业人才、专业精神”招商理念，大力推进全员招商，继续抓好驻点招商、产业链招商、园区招商，积极开展以商招商、商会招商、回归招商。主动对接国际国内500强、上市公司、央企、省企等旗舰型企业，千方百计引进一批大项目、好项目和区域总部，力争招商引资实际到位资金增长20%以上。坚持“筑巢”与“引凤”并重，加强园区软硬环境建设。坚持招商、安商同等重视，内商、外商公平对待，引智、引技同步推进。

加快项目建设。继续开展“项目建设年”活动，力争固定资产投资达到2300亿元。确保洪监高速公路、江北高速、荆松一级公路建成通车，加快推进沙公高速公路、石首长江大桥及连接线工程、煤炭铁水联运储配基地一期、公安朱家湾码头二期工程和松滋长江物流园等项目，抓好蒙华铁路项目对接服务，力争荆州机场开工建设。抓好沙隆达搬迁改造、沃特玛汽车电池、江陵煤电煤化、松滋荣成再生科技、公安循环造纸产业园、监利香港家居产业园、国家级淡水产品批发市场等重大项目。加快实施洞庭湖流域水污染防治项目，完成荆江大堤综合整治工程，续建荆南四河堤防加固工程，全力抓好洪湖分蓄洪区东分块工程和四湖流域防洪排涝灌溉工程。

强化服务保障。加大对项目建设的服务协调力度，切实提高项目的履约率、落地率和资金到位率。以“万名干部进万企”活动为载体，进一步完善“秘书制”、“企业直通车”、项目“绿色通道”、“一企一策”等服务，把更多的政府资源投入到服务实体经济的发展中去。扎实抓好政银企对接、产业链对接、产学研对接以及劳动力培训和用工对接，着力缓解企业融资难、开拓市场难、成果转化难和用工难。依法清退、盘活闲置用地，确保有限的土地资源效益最大化。

（三）突出改革开放与创新驱动。

加快重点领域改革。坚定不移地推进行政审批制度改革，最大限度地减少行政审批，建立全市统一、规范、高效的联审平台。加大中介机构清理整顿力度，重拳治理规划、测绘、设计、勘察、评估等中介机构垄断服务现象。深化财税体制改革，正税清费，降低企业税费负担，建立全口径预算管理、跨年度预算平衡机制和透明预算制度。深化投融资体制改革，大力发展天使投资、创业投资等各类创新投资机构，鼓励众筹、互联网金融等新型融资方式；进一步激活民间投资，通过政策支持、项目示范、PPP模式等，引导和鼓励社会资本以特许经营、参股控股等多种方式进入公共服务、产业发展、“双创”等领域。以管资本为主加强国有资产监管，着力解决国企、国资改革历史遗留问题。深化农村集体产权制度和国有农场改革，继续抓好事业单位分类改革、机关事业单位养老保险制度改革和教育、医疗、价格、户籍制度等民生领域改革。规范完善不动产统一登记。推进检验检测机构改革。完成公务用车制度改革。

提高对外开放水平。坚持“开放先导”战略，坚持内外需协调，着力提升城市、经济和社会各方面的对外开放度，大力促进外向型经济发展。积极打造全国区域级流通节点城市，谋划布局临空、临港、临铁产业园和物流园。加快荆州国家对外开放口岸、荆州开发区保税物流园的申报和建设，打造特色口岸，推进通关一体化、贸易安全便利化改革。坚持引进外资和扩大出口并重、“请进来”与“走出去”并举，创建国家级出口产品示范园区，推进企业参与国际经贸交流，扩大国际劳务输出规模。积极缔结国际友好城市。

增强科技创新能力。以创建国家创新型试点城市为抓手，深入实施创新驱动战略，强化企业创新主体地位，支持和引导企业加大产学研合作力度。建立市级创业创新扶持基金，建设一批院士专家工作站、创客中心和创新孵化基地。落实支持创业创新的财税金融政策，支持企业争创国家、省级重点实验室、工程技术研究中心，支持企业与高校、科研院所、中介服务组织形成技术创新战略联盟。加强新型智库建设。完善人才引进、培养、使用、激励机制，大力引进战略投资者、职业经理人、高新科技人才和海外研发团队。弘扬“筚路蓝缕、敢为人先”的精神，着力营造全社会尊崇“双创”、宽容失败的良好氛围。

（四）统筹城乡建设发展。

建好管好中心城区。增强凝聚力，提高首位度。启动新一轮城市规划修编工作，加大城市重点区域的城市设计力度，提高建筑设计水平，塑造城市特色。加强城建项目库建设，做好各项前期工作，确保中心城区总投资311亿元的城建项目按期推进。继续推进“滨长江、环古城”景观带建设，加快植物园、荆襄河湿地公园二期、玉桥公园二期建设。实施古城修复与保护世行贷款项目，抓好荆州职业技术学院扩建、荆州中心医院迁建、荆州中学与沙市中学新校区、新图书馆、规划档案馆等公共设施建设。启动城北快速通道和城市出入口建设，推进318国道城区段改线工程，改造升级荆州大道、景明观路、月堤路、护城河南路、金龙路等城市道路，加快沙北灌渠、排江工程、荆李线道路排水工程、东方大道截污干管工程等城市管网配套建设，逐步启动海绵城市建设。抓好政府购买棚改服务试点，加快推进棚户区、危房改造和老旧城区、城中村改造，引导建筑业、房地产业健康发展。开展“五城同创”活动，发动人民群众共治共管、共建共享。规范住宅小区物业管理。加强社区环境综合整治。改革城市管理综合执法，提高城市管理水平，着力解决交通拥堵、环境污染等“城市病”，打造“洁、绿、亮、美、齐、畅”的和谐宜居城市。

提升城乡一体化水平。落实中央城市工作会议提出的“紧凑集约、高效绿色”理念，强化全市规划一盘棋，统筹各类空间规划，推进“多规合一”。完善小城镇规划，强化管理职能，加快推进以人为核心的新型城镇化。坚持“产业先行”“一镇一业”，加快建设“四化同步”试点镇，支持松滋市推进国家新型城镇化综合试点，支持监利县白螺口子镇建设上升为省级战略。抓好“美丽乡村”和“荆楚派”民居特色建设示范，建设保留历史记忆、传统格局、山水风貌的美丽村镇。全面放开县级市和建制镇落户限制，落实流动人口居住证管理制度，依法维护进城农民土地承包权、宅基地使用权和集体收益分配权，促进有能力在城镇稳定就业和生活的农业转移人口举家进城落户，力争常住人口城镇化率提升1.5个百分点以上。

优化市域生态环境。坚持“生态立市”战略，加强污染防治、生态保护与生态建设，加快建设生态文明先行示范区。编制完成市域生态建设规划纲要，坚持源头严防、过程严管、后果严惩，着力防止无序、过度、分散开发导致生态破坏和环境污染。继续开展“三污同治”“三禁两治”，重点加强雾霾、水污染和土壤污染综合治理。继续推进“绿满荆州”行动，开展城镇绿地建设，加强洪湖、长湖、崇湖、洈水、天鹅洲等湿地保护修复，力争植树造林51万亩。加大生态环境保护工作对农村地区的覆盖，加强城乡垃圾无害化处理，提高乡镇污水处理厂运行效果。

（五）切实保障和改善民生。

打好脱贫攻坚战。坚持把“脱贫致富奔小康”作为“第一民生工程”来抓，严格落实专项扶贫预算，确保市级财政每年新增财政收入中用于扶贫开发的比例不低于15%。按照“六个精准”“五个一批”的总体要求，大力实施驻村帮扶、企业资助、能人带动、信贷撬动、技能培训、旅游扶贫等工程。建立健全县市党政主要负责人扶贫工作责任制考核办法，力争今年全市绝对贫困人口减少12.2万人。

抓好社会保障和社会事业。完善扶持创业促进就业政策，落实稳岗补贴、社保补贴、税费减免等政策，实施大学生就业促进计划、创业引领计划和农民工等群体返乡创业计划，积极引导和鼓励大学生、农民工等重点群体就业创业。加强社会保险扩面征缴，基本实现全民参保，全面建立城镇职工和城乡居民大病医疗保险制度。推进城乡社会救助体系建设，完善最低生活保障、特困人员供养、残疾人扶助等兜底性制度。继续实施“农村义务教育薄弱学校全面改造计划”，加快城乡义务教育公办学校标准化建设，通过教育信息化扩大优质教育覆盖面。鼓励发展普惠性学前教育，加快普及高中阶段教育，推进职业教育创新发展，支持长江大学等在荆高校加快建设一流大学和一流学科。深入开展全民健身活动，支持体育场馆市场化运营。高度重视文化遗产的保护传承利用，抓好基层文化阵地建设，加强文艺精品创作生产。健全公办养老、居家养老、社区养老服务网络。落实“全面二孩”政策，加强出生人口性别比综合治理，促进区域人口可持续均衡发展。加快推进洪湖渔民上岸安居工程。认真抓好国防动员、民兵预备役和人民防空工作，支持驻荆部队、武警部队建设，进一步做好外事侨务、台湾事务、民族宗教、防震减灾、对口支援、邮政通信、统计、移民、农垦、烟草、盐务、供销、气象、史志、保密和无线电等工作。

加强和创新社会治理。全面启动“七五”普法，增强全社会的法治意识。完善矛盾纠纷协调化解机制，将信访工作导入法治化轨道，妥善处理好土地征用、拆迁安置、金融信贷、环境污染等方面的矛盾纠纷。完善立体化治安防控体系，实施群防群治和网格化管理。加强网络舆情引导管理，健全网络突发事件处置机制。深入推进“餐桌污染”治理，建设“食品放心工程”，构筑食品药品安全社会共治防线。深入推进平安荆州建设，加强安全生产、道路交通、消防等社会公共安全监管和城市应急体系建设，始终保持打击非法集资和毒品犯罪高压态势，控制和减少较大以上事故，坚决遏制重特大公共安全事故发生，争创全国社会治安综合治理“长安杯”。

扎实办好民生实事。一是新增城镇就业70000人，扶持创业5000人，带动就业20000人，就业创业培训5000人，发放创业担保贷款2亿元。二是开工建设保障性住房44968套，基本建成18781套，分配入住11720套，新增租赁补贴440户。三是中心城区购置100台新能源绿色公交车。四是建设一批放心早餐示范店，完成中心城区5家农贸市场升级改造。五是在全市240所小学建设“希望厨房”。六是免费为100名贫困下肢残疾人安装假肢，为300名贫困白内障患者实施复明手术，为600名贫困残疾儿童实施抢救性康复。七是为经济困难群众和农民工办理法律援助案件2600件，办理法律援助事项26000件以上。八是免费培训新型职业农民4000人，建设100个省级绿色示范乡村。九是对500个符合“雨露计划”职业教育对象条件的贫困家庭子女，每年给予3000元补助。十是关停中心城区20家“十五小”企业。

（六）全面加强政府自身建设。

坚持依法行政。自觉运用法治思维和法治方式深化改革、推动发展、化解矛盾、维护稳定，营造办事依法、遇事找法、解决问题用法、化解矛盾靠法的良好法治环境。自觉接受党内监督、人大监督、民主监督、司法监督，完善舆论监督和社会监督机制，认真办理人大代表建议和政协提案，主动听取民主党派、工商联、无党派人士和人民团体的意见和建议。支持人大依法行使地方法规制定权，坚持重大事项提请人大决策，坚持民主协商于决策之前、决策之中。推进政府及部门职责法定化，严格依照法定权限和程序行使权力。规范行政执法自由裁量权，做好规范性文件的合法性审查。全面推进政务公开，坚持以公开为常态、不公开为例外，推进决策公开、执行公开、管理公开、服务公开、结果公开，提高政府工作的透明度和公信力。

坚持改革创新。认真落实国家简政放权、放管结合、优化服务等各项改革政策，推进政府职能转变。进一步强化执政为民的理念，以公众需求为导向提供政务服务，以公众评价为标准改进政务服务，不断提高政府精细化管理水平。进一步创新发展思路，摒弃以浪费土地资源、牺牲生态环境为代价的发展方式，从供需两端发力，推进结构性改革，在做大总量、做多增量、做优质量上找准新路子，实现新突破。进一步优化服务方式，加快政务系统标准化与信息化建设，提升“大数据”综合运用能力，让信息多跑路，让群众少跑腿。

坚持务实担当。严格绩效管理，鼓励竞进作为，引导广大干部投身招商引资、项目建设、征迁安置、信访维稳、脱贫攻坚、城市创建等工作一线。加强督查考核，狠抓执行落实，建立重大事项、重大决策定向督办制度，做到事事有着落、件件有回音。加强重点工作调度，抓进度、排位次，促进比学赶超、竞相发展。

坚持严以用权。必须时刻牢记权力来自于人民，公权必须公用，分清公与私。必须认真落实“主体责任”“一岗双责”，坚持“力度统一论”，深入推进党风廉政建设和反腐败斗争，努力营造“干部清正、政府清廉、政治清明”的政治生态。巩固“三严三实”专题教育成果，推动政风作风建设不断深入。加强行政监察和审计监督，严格公共资金、公共资源和国有资产监管。强化“治庸问责”，重点惩治“不作为、乱作为、慢作为、懒作为”问题，形成“对党忠诚、敢于担当、干净干事、风清气正”的干事创业氛围。

各位代表！荆州正处在改革攻坚、爬坡过坎的关键时期，让我们在省委、省政府和市委的正确领导下，坚定发展信心，升腾发展气场，履职尽责，扎实工作，为实现壮腰工程“五年大跨越、十年大振兴”而努力奋斗！
